# Gelsolin-Like Domain 3 Plays Vital Roles in Regulating the Activities of the Lily Villin/Gelsolin/Fragmin Superfamily

**DOI:** 10.1371/journal.pone.0143174

**Published:** 2015-11-20

**Authors:** Dong Qian, Qiong Nan, Yueming Yang, Hui Li, Yuelong Zhou, Jingen Zhu, Qifeng Bai, Pan Zhang, Lizhe An, Yun Xiang

**Affiliations:** 1 MOE Key Laboratory of Cell Activities and Stress Adaptations, School of Life Sciences, Lanzhou University, Lanzhou, 730000, China; 2 College of Chemistry and Chemical Engineering, Lanzhou University, Lanzhou, 730000, China; Hainan University, CHINA

## Abstract

The villin/gelsolin/fragmin superfamily is a major group of Ca^2+^-dependent actin-binding proteins (ABPs) involved in various cellular processes. Members of this superfamily typically possess three or six tandem gelsolin-like (G) domains, and each domain plays a distinct role in actin filament dynamics. Although the activities of most G domains have been characterized, the biochemical function of the G3 domain remains poorly understood. In this study, we carefully compared the detailed biochemical activities of ABP29 (a new member of this family that contains the G1-G2 domains of lily ABP135) and ABP135_G1-G3_ (which contains the G1-G3 domains of lily ABP135). In the presence of high Ca^2+^ levels *in vitro* (200 and 10 μM), ABP135_G1-G3_ exhibited greater actin severing and/or depolymerization and nucleating activities than ABP29, and these proteins had similar actin capping activities. However, in the presence of low levels of Ca^2+^ (41 nM), ABP135_G1-G3_ had a weaker capping activity than ABP29. In addition, ABP29 inhibited F-actin depolymerization, as shown by dilution-mediated depolymerization assay, differing from the typical superfamily proteins. In contrast, ABP135_G1-G3_ accelerated F-actin depolymerization. All of these results demonstrate that the G3 domain plays specific roles in regulating the activities of the lily villin/gelsolin/fragmin superfamily proteins.

## Introduction

The villin/gelsolin/fragmin superfamily is an important class of multifunctional actin-binding proteins (ABPs) that regulate dynamic remodeling of the actin cytoskeleton via actin nucleating, severing, capping and bundling activities in eukaryotes [1–3]. These superfamily proteins are typified by the possession of three or six G domains [1,4,5]. In mammals and bacteria, each member of this superfamily is encoded by distinct genes, such as villin, which contains six G domains and one head piece domain [6,7]; gelsolin, which contains six G domains [1,4]; fragmin/severin, which has three G domains [8]; and GSNL-1 in *Caenorhabditis elegans*, which contains four G domains [9]. However, although the genes enconding villin have been reported in detail[10–12], gelsolin-like proteins in plants have only been mentioned in a few reports [12,13]. The first two plant villins to be identified were ABP135 [14,15] and ABP115 [16,17], which were isolated from lily pollen. In addition, there are six villin homologs in *Oryza sativa* and five in *Arabidopsis thaliana* [10,18]. Recently, two new members were identified: PrABP80 [13], which was isolated from *poppy* pollen and contains six G domains, and LdABP41 [19], which was isolated from *Lilium davidii* pollen and contains the G1-G3 domains. Interestingly, the smallest identified member of the superfamily is ABP29 [20], which contains only the G1 and G2 domains and part of the G2-G3 linker from *Lilium* pollen and is an alternative splicing product of plant ABP135 [20,21].

Despite sharing conserved protein sequences and three-dimensional structures, each G domain of the villin/gelsolin/fragmin superfamily plays a distinct role in actin dynamics, endowing the proteins with multifunctional and distinct activities [1,12]. Through several decades of research, the biochemical functions of most G domains in this superfamily have been well characterized. For example, the N-terminal 17-kD chymotryptic segment (CT14N) that retains weak actin-binding functions was described as the G1 domain, which can bind to monomeric actin in a Ca^2+^-insensitive manner [22]. The truncation of gelsolin with G1 domain and 10 extra amino acids from the start of the G2 domain has weak F-actin severing activity, but this severing activity of the truncation is only one percent of the full gelsolin protein [23]. The G2 domain contains an F-actin binding site, which also has actin capping activity [24]. In addition, the G2 domain has a phosphatidylinositol 4,5-bisphosphate (PIP_2)_ binding site and binds to tropomyosin in a Ca^2+^- and pH-sensitive manner [24,25]. Further, the G2 domain of GSNL-1 functions as a regulatory domain for Ca^2+^-dependent conformational changes [26]. Similar to the G1 domain, the G4 domain also binds to monomeric actin [5,27] In addition, it is generally accepted that the C-terminus of gelsolin acts as a helix latch and binds the G2 domain to maintain gelsolin in a Ca^2+^-free compact state [5,28,29]. However, the biochemical function of the G3 domain is still poorly understood beyond its role as a spacer.

We have previously demonstrated that both ABP29 and LdABP41 accelerate actin nucleation, severing and capping of actin filaments in a Ca^2+^- and/or PIP_2_-regulated manner *in vitro* [19,20,30]. Surprisingly, significant differences between ABP29 and LdABP41 and other gelsolin-like proteins have been shown in dilution-mediated F-actin depolymerization assays [20,30]. Specifically, ABP29 inhibits F-actin depolymerization, whereas LdABP41 accelerates F-actin depolymerization similar to other typical members of this superfamily. Why is this? Does the loss of the G3 domain cause this difference? Unfortunately, the gene encoding LdABP41 has not been cloned, although mass spectrometry analysis has shown that LdABP41 shares substantial similarity with lily ABP135 and that it may possess the full G1-G3 domains [19,30]. Thus, it remains unclear whether LdABP41 contains a complete G3 domain and whether the G3 domain causes the observed difference between ABP29 and LdABP41.

Here, we cloned the G1-G3 domains of ABP135 (and designated them as ABP135_G1-G3_), which behaves similarly to LdABP41 [19,30] *in vitro*. We then carefully compared the activities of ABP29 and ABP135_G1-G3_ in regulating actin dynamics. Our results demonstrated that the G3 domain endows ABP135_G1-G3_ with greater actin severing and/or depolymerization and nucleating abilities than ABP29 in the presence of high Ca^2+^ but that its actin capping activity is weaker than that of ABP29 in the presence of low Ca^2+^. These findings shed new light on the functions of the G3 domain.

## Materials and Methods

### Expression and purification of recombinant proteins

The cDNA coding sequence (CDS) of ABP135_G1-G3_ was amplified from lily flowers, and ABP29_G1-G2_ and ABP29-△C6 were amplified from pGEX-4T-ABP29 [20]. The primers used for cloning are described in the Table 1. To obtain recombinant proteins, ABP135_G1-G3_, ABP29_G1-G2_ and ABP29-△C6 were cloned into the pGEX-4T vector and then expressed in the *E*. *coli* BL21 (DE3) strain by induction with 0.5 mM isopropylthio-β-D-galactopyranoside (IPTG) overnight at 26°C according to previously described methods [31]. Next, recombinant proteins were then affinity-purified using glutathione–Sepharose 4B resin (GE Healthcare) according to the manufacturer’s instructions. The purified proteins were dialyzed against modified buffer A3 (10 mM Tris-HCl, 7 mM PIPES, 200 μM CaCl_2_, 0.5 mM DTT and 0.2 mM ATP, pH 7.0).

**Table 1:** Primers used in this study.

Primer name	Sequence (5’–3’)
ABP135_G1-G3_-F	GAATTCATGGCCAACTCTTCAAAAAAT
ABP135_G1-G3_-R	GTCGACAGACTCAAAGTTGGACTTAAA
ABP29-F	GAATTCATGGCCAACTCTTCAAAAAAT
ABP29-G1-2-R	GTCGACTCAACCTGACCCAGATTCTGCTG
ABP29-linker-R	GTCGACTCAATAAAGCTTCCCTGGCGTGG

The restriction enzyme sites are underlined.

"(F)" indicates the forward primer and

"(R)" indicates the reverse primer.

According to previously described methods [32,33], actin was isolated from rabbit skeletal muscle acetone powder and labeled on Cys^374^ with pyrene iodoacetamide. Human recombinant profilin I was purified as described previously [34]. Prior to all experiments, all recombinant proteins were preclarified by centrifugation at 100,000 g for 1 h at 4°C.

### High-speed co-sedimentation assay

A high-speed co-sedimentation assay was performed as described by Xiang et al. [20]. Preformed F-actin (5 μM) was incubated with different concentrations of recombinant proteins in the presence of various concentrations of free Ca^2+^ at 22°C for 1 h. The free Ca^2+^ concentration was calculated with EGTA software by Petesmif (http://pcwww.liv.ac.uk/~petesmif/petesmif/software/webware06/EGTA/EGTA.htm), as described in Khurana et al. [35]. According to our calculations, 2 mM, 1 mM, 0.5 mM and 0.2 mM EGTA were equivalent to the volumes of 0.041 μM, 0.091 μM, 0.243 μM and 10 μM free Ca^2+^, respectively, in all of our experimental systems. The samples were centrifuged at 100,000 g for 1 h at 4°C (CS120GXII, Hitachi). Fractions containing the supernatants and pellets were resolved using SDS-PAGE, and the gels were stained with Coomassie Brilliant Blue R 250. The amounts of actin in the supernatants and pellets were analyzed using Quantity One software (version 4.6.2; Bio-Rad).

### Direct observation of actin filaments by fluorescence microscopy

Microscopic observation of actin filament severing was performed as described previously [20,36]. Preformed F-actin (5 μM) was incubated with 100 or 200 nM ABP29 or ABP135_G1-G3_ in the presence of various concentrations of free Ca^2+^ at room temperature for 30 min, and the reaction mixtures were labeled with Alexa 488-phalloidin (Molecular Probes Invitrogen), as previously described [6]. Then, the reaction mixtures were diluted to 60 nM with F buffer (10x F buffer: 500 mM KCl, 10 mM MgCl_2_, 10 mM Tris-HCl, 200 μM CaCl_2_ and 100 mM ATP, pH 7.0) and 1 μl of each diluted sample was placed on a nitrocellulose-coated coverslip. The samples were observed using a confocal laser-scanning microscope (Olympus FV-300) mounted on a fluorescence microscope (Olympus IX-70) with a 100x oil-immersion objective, and the images were captured using Olympus Fluoview 4.0 software and processed with Adobe Photoshop CS2. Filament lengths were measured using Image J software.

### Actin filament severing assay

An actin filament severing assay was performed as described previously [37] with modifications as follows: 2 μM CapG-actin (1:200) filaments were generated by polymerization overnight at 25°C in S2 buffer (0.5 mM ATP, 1 mM DTT, 200 μM CaCl_2_, 0.1 mM KCl, 1 mM MgCl_2_, and 10 mM Tris-HCl, pH 7.5) with 5% pyrene-labeled actin and CapG (purified from recombinant protein expressed in *E*. *coli* BL21). Then, the addition of S2 buffer diluted actin filaments to 100 nM with varying concentrations of ABP29 or ABP135_G1-G3_, and immediately the decrease in pyrene fluorescence intensity that accompanied actin depolymerization was monitored for 300 s.

### Actin nucleation assay

Actin nucleation was performed as described previously [20]. Monomeric actin (5 μM 5% pyrene-labeled) was incubated with various concentrations of ABP29 or ABP135_G1-G3_ at room temperature for 5 min in the presence of 200 μM, 10 μM or 0.041 μM free Ca^2+^. Pyrene fluorescence was monitored for 100 s with a Fluoro-Max-4^®^ spectrofluorometer (HORIBA Jobin Yvon) immediately after the addition of one-tenth volume of 10x F buffer.

### Actin filament elongation assay

An elongation assay was performed as described previously [20,38]. Prepared actin filament seeds (0.4 μM) were incubated with different concentrations of ABP29 or ABP135_G1-G3_ in the presence of different Ca^2+^ concentrations at room temperature for 5 min and then supplemented with 1 μM G-actin (5% pyrene-labeled) saturated with 4 μM human profilin I and one-tenth volume of 10x F buffer. After actin elongation was initiated, the change in pyrene fluorescence accompanying actin polymerization was monitored.

### Critical concentration determination

According to a previously described method [9], pyrene-labeled G-actin (5% labeled) at varying concentrations (0–4 μM) was polymerized for 18 h at room temperature in the presence of 0.5 μM ABP29 or ABP135_G1-G3_ in F buffer, and the final fluorescence intensity of pyrene (excitation at 365nm and emission at 407 nm) was measured.

### Actin filament depolymerization assay

Dilution assays were performed according to a previously described method [20]. Briefly, various concentrations of the recombinant proteins were incubated with 5 μM preformed F-actin (50% pyrene-labeled) in the presence of different concentrations of free Ca^2+^ for 5 min at room temperature. The decrease in pyrene fluorescence intensity that accompanied actin depolymerization was monitored for 400 s after the solution was diluted 25-fold with G buffer (5 mM Tris-HCl, 0.2 mM ATP and 0.5 mM DTT, pH 8.0) containing various concentrations of CaCl_2_.

### Statistical analysis

Statistical analyses were conducted using SPSS 16.0 followed by Student’s *t* test. At least three independent experiments were performed.

### Accession numbers

The sequence data from this article can be found in the GenBank/EMBL databases under the following accession numbers: ABP29 (EF042093), ABP135 (AAD54660), Human cap G (NP _001243068.1) and Human Gelsolin (NM_198252).

## Results

### The actin severing and/or depolymerization activity of ABP135_G1-G3_ is stronger than that of ABP29 in the presence of high concentrations of calcium

**Fig 1:**
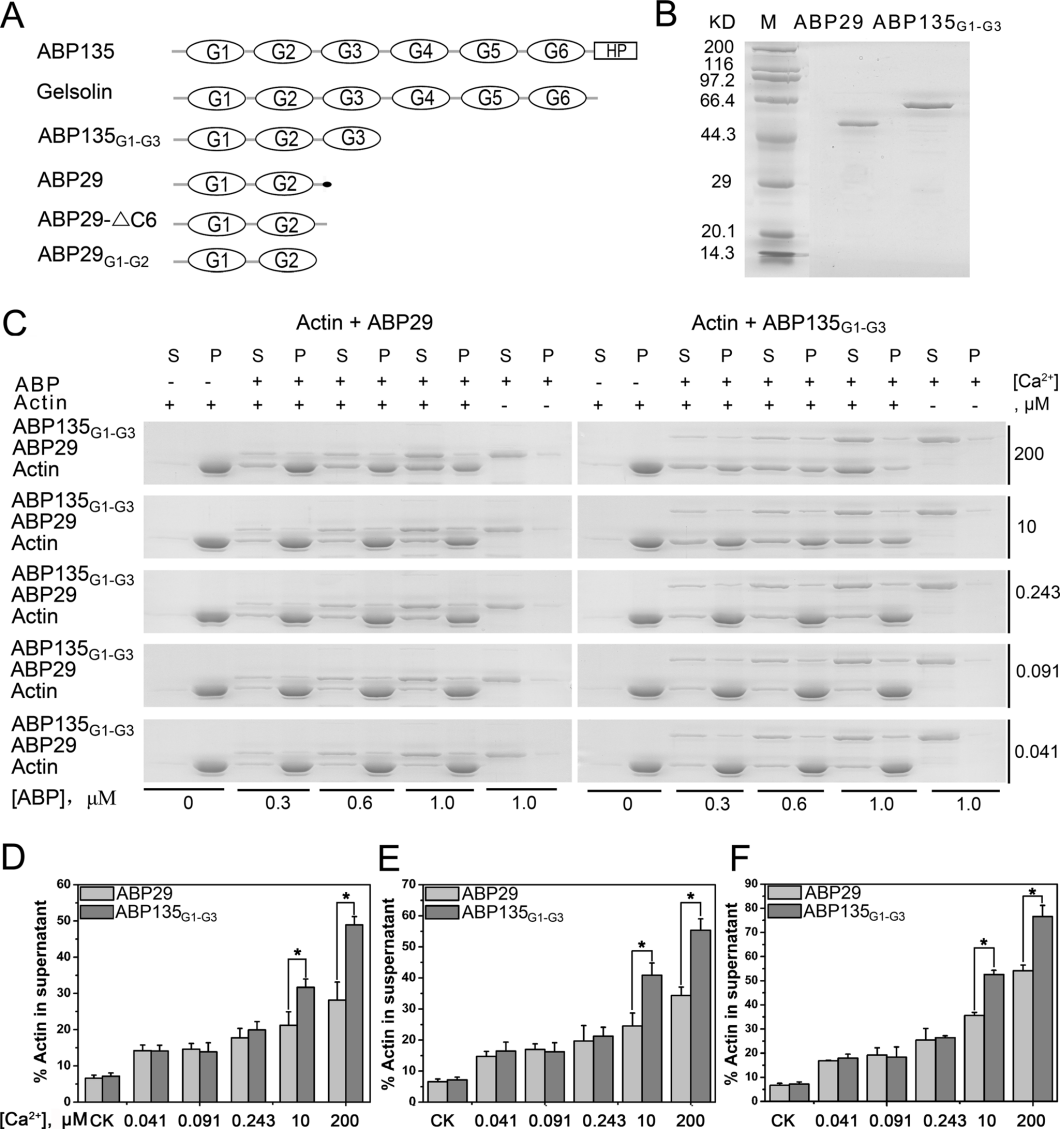
Recombinant ABP29 and ABP135_G1-G3_ fusion proteins sever and/or depolymerize actin filaments in a calcium-dependent manner. (A) Domain structures of gelsolin superfamily members studied in the paper. The lily ABP135,with six G domains and a headpiece (HP) domain at the C-terminus, is designated as ABP135. Full-length human gelsolin, containing six G-domains, is designated as Gelsolin. ABP135_G1-G3_ containing the G1-G3 domains is part of ABP135. *Lilium* ABP29 is a splicing product of ABP135, and the small black dot at the end of ABP29 represents the six amino acids that differ from ABP135. ABP29-△C6 has six fewer amino acids than ABP29 at the C-terminus. ABP29_G1-G2_ contains only the G1-G2 domains of ABP29. (B) SDS-PAGE analysis of purified recombinant ABP29 and ABP135_G1-G3_. Molecular mass markers (M) in kiloDaltons are shown to the left of the gel. (C) Detailed comparison of ABP29 and ABP135_G1-G3_ severing and/or depolymerization activities in the presence of different concentrations of Ca^2+^ with high-speed co-sedimentation assays; "(s)" indicates supernatant, "(p)" indicates pellets, and "ABP" indicates ABP29 or ABP135_G1-G3_. (D-F) Statistical analysis results from (C). The graph shows the percentage of actin in the supernatant. The concentrations of the recombinant proteins are 0.3, 0.6 and 1.0 μM, respectively in (D-F). The CK is the percentage of actin in the supernatant in the absence of ABP. The error bars represent ± SE (n = 3); *P < 0.05 (Student’s *t* test).

To identify whether the G3 domain has an effect on the activities of villin/gelsolin/fragmin superfamily members, we generated different truncated variants of lily ABP135 and ABP29 using deletion mutagenesis (Fig 1A **)**. Initially, we purified recombinant GST-ABP29 and GST-ABP135_G1-G3_ (0–342 AA) to test the function of the G3 domain in F-actin severing and/or depolymerization (Fig 1B). According to previous studies, the growing pollen tube possesses a "tip-focused" gradient of free Ca^2+^, in which the cytosolic concentration of free Ca^2+^ ranges from 2–10 μM (in the apical region) to 20–200 nM (in the shank region) [39–41]; therefore, we selected Ca^2+^ concentrations for our experiments that corresponded to *in vivo* function. The specific protein concentrations for our experiments were chosen based on previous work [19,20,30]. A high-speed co-sedimentation assay was performed in the presence of 0.3, 0.6 and 1 μM ABP29 or ABP135_G1-G3_. In the presence of 200 μM Ca^2+^, the presence of ABP29 or ABP135_G1-G3_ caused a significant amount of actin to be redistributed to the supernatant in a dose-dependent manner (Fig 1C–1F), in agreement with our previous studies [19,20]. In addition, in the presence of high concentrations of Ca^2+^ (200 μM and 10 μM), ABP135_G1-G3_ increased the amount of supernatant actin to a greater degree than ABP29 did. In the presence of low concentrations of Ca^2+^, such as 243 nM, 91 nM and 41 nM, there were no obvious differences in the amounts of supernatant actin between the two proteins (Fig 1C, 1D and 1E).

**Fig 2:**
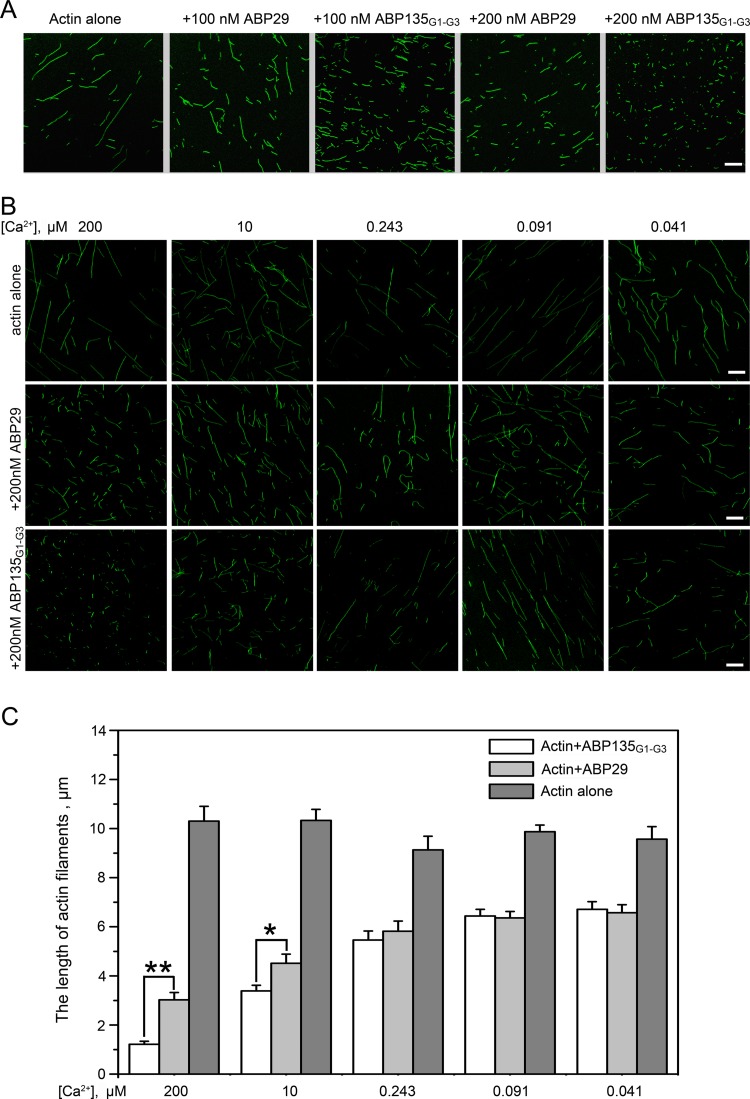
Direct visualization of actin filament severing and/or depolymerization by recombinant ABP29 and ABP135_G1-G3_. (A) Direct observation of the severing and/or depolymerization activities of ABP29 and ABP135_G1-G3_ at different concentrations (100, 200 nM) in the presence of 200 μM free Ca^2+^. The scale bar is 10 μm. (B) Direct observation of the severing and/or depolymerization activities of 200 nM ABP29 and ABP135_G1-G3_ in the presence of various concentrations (200, 10, 0.243, 0.091 and 0.041 μM) of free Ca^2+^. The scale bar is 10 μm. (C) Quantification of actin filament length from the micrographs from (B). The lengths of actin filaments were measured by Image J. Thirty pictures for each condition were analyzed (25–70 actin filaments in every picture), and quantification of 10 pictures represents a single result. Error bars represent ± SE (n = 3). *P < 0.05, **P < 0.01 (Student’s *t* test).

To further confirm the above results, fluorescence microscopy was employed to directly visualize Alexa 488–phalloidin-labeled actin filaments (Fig 2). As shown in Fig 2A, compared with actin alone (average lengths = 9.75 ± 1.94 μm), 100 nM and 200 nM ABP29 significantly reduced the lengths of actin filaments (average lengths = 4.05 ± 0.94 μm and 2.81 ± 0.83 μm, respectively), and 100 nM and 200 nM ABP135_G1-G3_ resulted in even shorter actin filaments (average lengths = 2.23 ± 0.83 μm and 1.05 ± 0.34 μm, respectively). These results indicate that ABP135_G1-G3_ possesses stronger severing and/or depolymerization activity than ABP29 in the presence of 200 μM free Ca^2+^ (Fig 2A). Furthermore, the F-actin severing and/or depolymerization activities of ABP29 and ABP135_G1-G3_ were observed when the Ca^2+^ concentration was changed using EGTA (Fig 2B and 2C). In the presence of 200 μM and 10 μM free Ca^2+^, ABP135_G1-G3_ caused greater amounts of smaller fragmentation of actin filaments compared with ABP29. However, when Ca^2+^ was chelated by EGTA, resulting in a reduction in the concentration (for example, 0.243, 0.091 or 0.041 μM), there was no significant difference in actin filament length between ABP29 and ABP135_G1-G3_, which is consistent with the co-sedimentation assay results described above.

**Fig 3:**
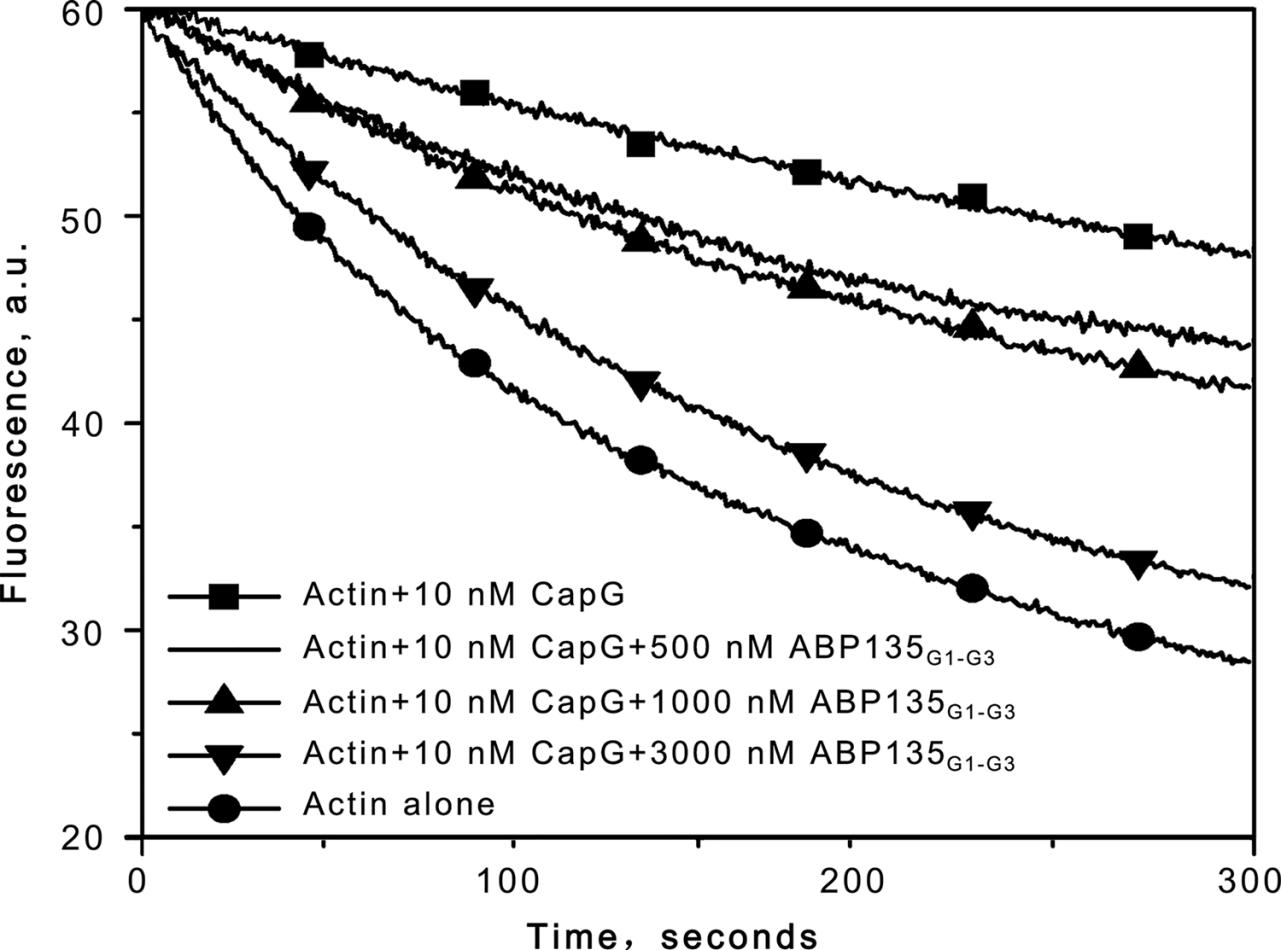
Effects of ABP135_G1-G3_ on actin filament severing. A total of 2 μM preformed CapG capped pyrene actin filaments was diluted to 100 nM in S2 buffer with various concentrations of ABP135_G1-G3_, and immediately, the decrease in pyrene fluorescence intensity that accompanied actin depolymerization was monitored for 300 s.

According to the previous work by Xiang et al. (2007), the severing activity of ABP29 was determined by a time course of actin filament fragmentation [20]. To determine whether the severing by ABP135_G1-G3_ truly takes place, an actin filament severing assay was performed. As shown in Fig 3, ABP135_G1-G3_ severed actin filaments. Collectively, these results demonstrate that the existence of the G3 domain confers ABP135_G1-G3_ with greater F-actin severing and/or depolymerization ability in the presence of high concentrations of Ca^2+^.

### ABP135_G1-G3_ exhibits more effective actin nucleating activity than ABP29 in the presence of high levels of Ca^2+^

**Fig 4:**
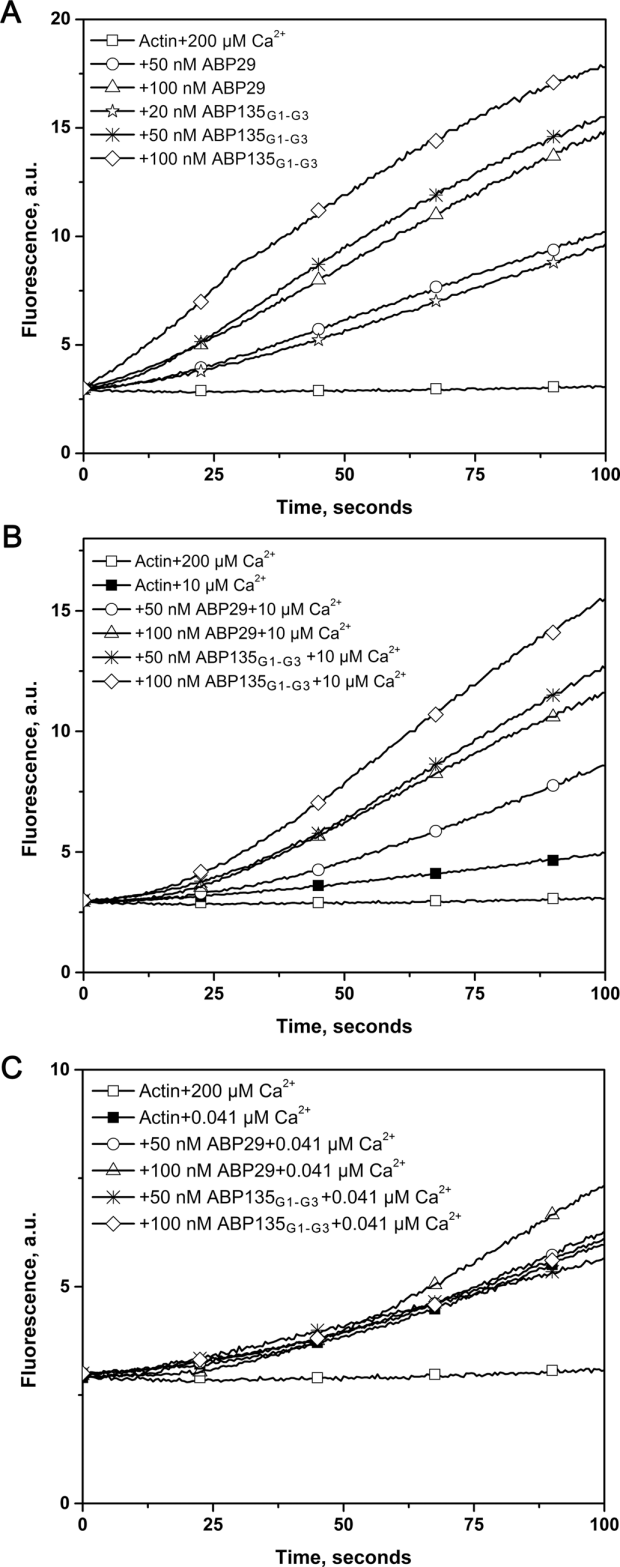
Recombinant ABP29 and ABP135_G1-G3_ nucleate actin polymerization. (A) Time course of the change in pyrene fluorescence accompanying actin polymerization in the presence of ABP29 or ABP135_G1-G3_. Increasing amounts of ABP29 or ABP135_G1-G3_ were added to 5 μM G-actin (5% pyrene-labeled) in the presence of 200 μM free Ca^2+^. Different combinations of reactions are indicated in the upper left. (B) and (C) In the presence of 10 μM free Ca^2+^ and 0.041 μM free Ca^2+^ respectively, time course of the change in pyrene fluorescence accompanying actin polymerization in the presence of ABP29 or ABP135_G1-G3_. Different combinations of reactions are indicated in the upper left.

To test the effect of the G3 domain on actin polymerization dynamics, pyrene fluorescence was used to monitor rabbit muscle actin polymerization kinetics (Fig 4). Both ABP29 and ABP135_G1-G3_ diminished the lag period corresponding to the nucleation step for actin polymerization in a concentration-dependent manner in the presence of 200 μM or 10 μM free Ca^2+^ (Fig 4A and 4B), but the activity of ABP135_G1-G3_ was higher than that of ABP29. However, when Ca^2+^ was chelated by EGTA, the nucleation activities of ABP29 and ABP135_G1-G3_ were significantly decreased, and the difference in the nucleating activity between ABP135_G1-G3_ and ABP29 was minor in the presence of low levels of Ca^2+^. These results suggest that the G3 domain regulates the actin nucleating activities of ABP135_G1-G3_ and ABP29 in the presence of different concentrations of Ca^2+^.

### The capping effect of ABP29 is stronger than that of ABP135_G1-G3_ in the presence of low Ca^2+^

**Fig 5:**
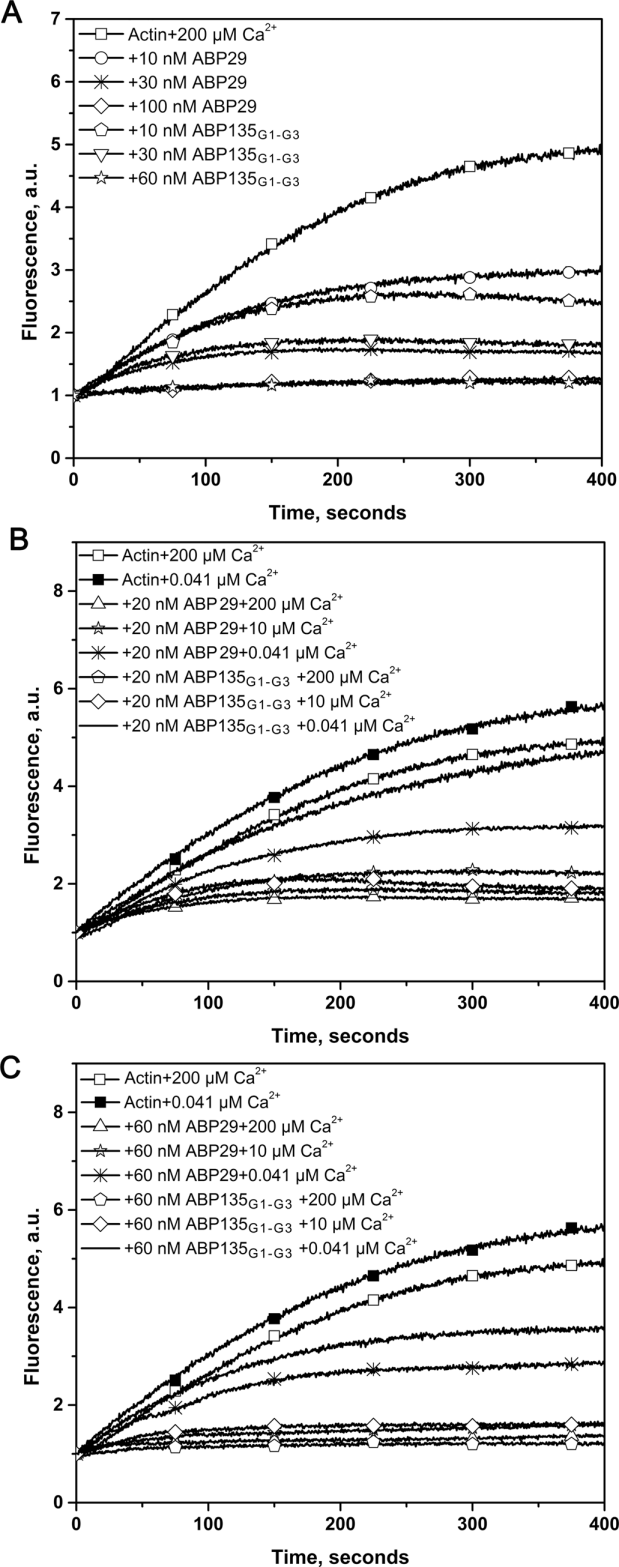
Recombinant ABP29 and ABP135_G1-G3_ inhibit actin filament elongation. (A) Kinetics of actin filament barbed end elongation with various concentrations of ABP29 or ABP135_G1-G3_ in the presence of 200 μM free Ca^2+^. Different combinations of reactions are indicated in the upper left. (B) and (C) Kinetics of actin filament barbed end elongation with 20 nM and 60 nM ABP29 or ABP135_G1-G3,_ respectively, in the presence of different concentrations of free Ca^2+^. Different combinations of reactions are indicated in the upper left.

We examined the effects of ABP29 and ABP135_G1-G3_ on the critical concentration (Cc) of actin to determine whether either of these proteins caps the barbed ends. As shown in S1A Fig, the Cc was shifted in the presence of ABP29 or ABP135_G1-G3_, and quantitative analyses demonstrated that ABP29 increased the Cc from 0.23 ± 0.03 μM for actin filaments with free barbed ends to 0.37 ± 0.01 μM, while ABP135_G1-G3_ shifted the Cc to 0.72± 0.02 μM. This shift may have resulted from G-actin binding. Then, the barbed ends of actin filaments were capped with CapG, and ABP29 was found to increase the Cc of actin filaments from 0.51 ± 0.03 μM to 0.69 ± 0.01 μM, while ABP135_G1-G3_ shifted the Cc to 0.85± 0.02 μM. These results (S1B Fig) indicate that both ABP29 and ABP135_G1-G3_ could bind G-actin. Seed elongation assays were subsequently performed to examine whether ABP29 and ABP135_G1-G3_ exhibit similar activities in inhibiting actin elongation from the barbed ends. As shown in Fig 5A, ABP29 and ABP135_G1-G3_ could suppress F-actin elongation effectively in the presence of high concentrations of free Ca^2+^ (200 μM and 10 μM). However, when free Ca^2+^ was chelated by EGTA to 0.041 μM, the capping activity of ABP135_G1-G3_ was arrested more dramatically than ABP29, and ABP29 had a stronger inhibitory effect on actin elongation than ABP135_G1-G3_ (Fig 5B and 5C). Taken together, these data suggest that the inhibitory effects of ABP135_G1-G3_ are more sensitive to Ca^2+^ than those of ABP29 due to its extra G3 domain relative to ABP29 and that the capping activity of ABP29 is stronger than that of ABP135_G1-G3_ in the presence of low Ca^2+^ concentrations.

### ABP29 inhibits F-actin depolymerization, whereas ABP135_G1-G3_ accelerates its depolymerization during the process of dilution-mediated depolymerization

**Fig 6:**
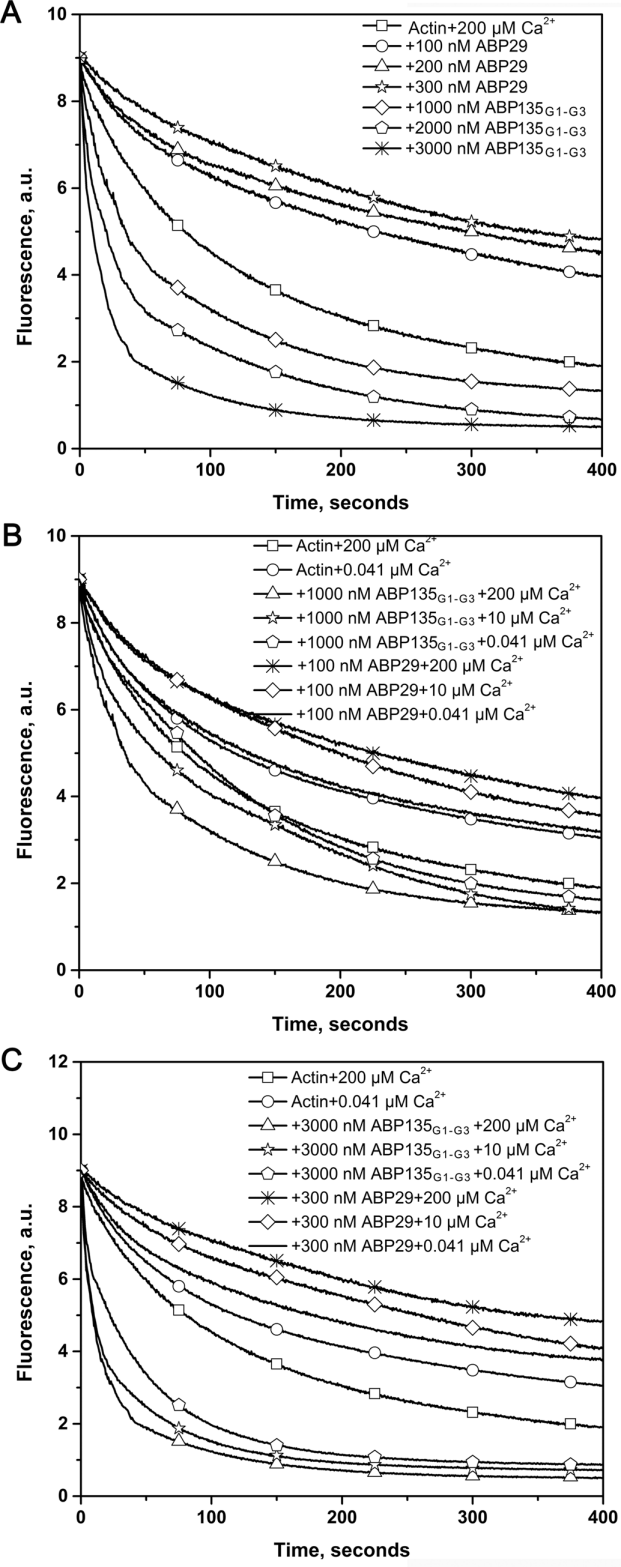
Recombinant ABP29 and ABP135_G1-G3_ exert opposing effects on the actin dynamics of depolymerization. (A) Kinetics of actin depolymerization in the presence of various concentrations of ABP29 or ABP135_G1-G3_ in the presence of 200 μM free Ca^2+^. The change in pyrene fluorescence accompanying actin depolymerization was monitored. Different combinations of reactions are indicated in the upper right. (B) and (C) Fixed concentrations of ABP29 or ABP135_G1-G3_ were incubated with 5 μM F-actin (50% pyrene-labeled) in the presence of free Ca^2+^ chelated by EGTA. The change in pyrene fluorescence accompanying actin depolymerization was monitored. Different combinations of reactions are indicated in the upper right.

Recent studies have demonstrated that ABP29 inhibits F-actin depolymerization in a dilution-mediated actin depolymerization assay [20], whereas LdABP41 promotes actin depolymerization [30]. However, it is not clear whether these differences are caused by the G3 domain because the coding gene for LdABP41 is unknown [19]. Therefore, the same assay was performed to verify the function of the G3 domain in actin dynamics. The results showed that ABP29 stabilized F-actin by inhibiting actin depolymerization in a Ca^2+^-dependent manner during the process of dilution-mediated depolymerization, as indicated by previous studies [20] (Fig 6A–6C), whereas ABP135_G1-G3_ accelerated F-actin depolymerization in a manner similar to LdABP41, except that LdABP41 possesses higher activity [30]. In addition, as shown in Fig 6B and 6C, the process of depolymerization by ABP135_G1-G3_ can be divided into two phases, similar to the function of G1-G3 domains of gelsolin [42]. During the early phase, ABP135_G1-G3_ may sever actin filaments to generate greater numbers of filament ends and accelerate F-actin depolymerization in a Ca^2+^-sensitive manner. All of the results described above demonstrate that the G3 domain plays a vital role in regulating ABP135_G1-G3_ relative to ABP29 during the process of dilution-mediated depolymerization.

**Fig 7:**
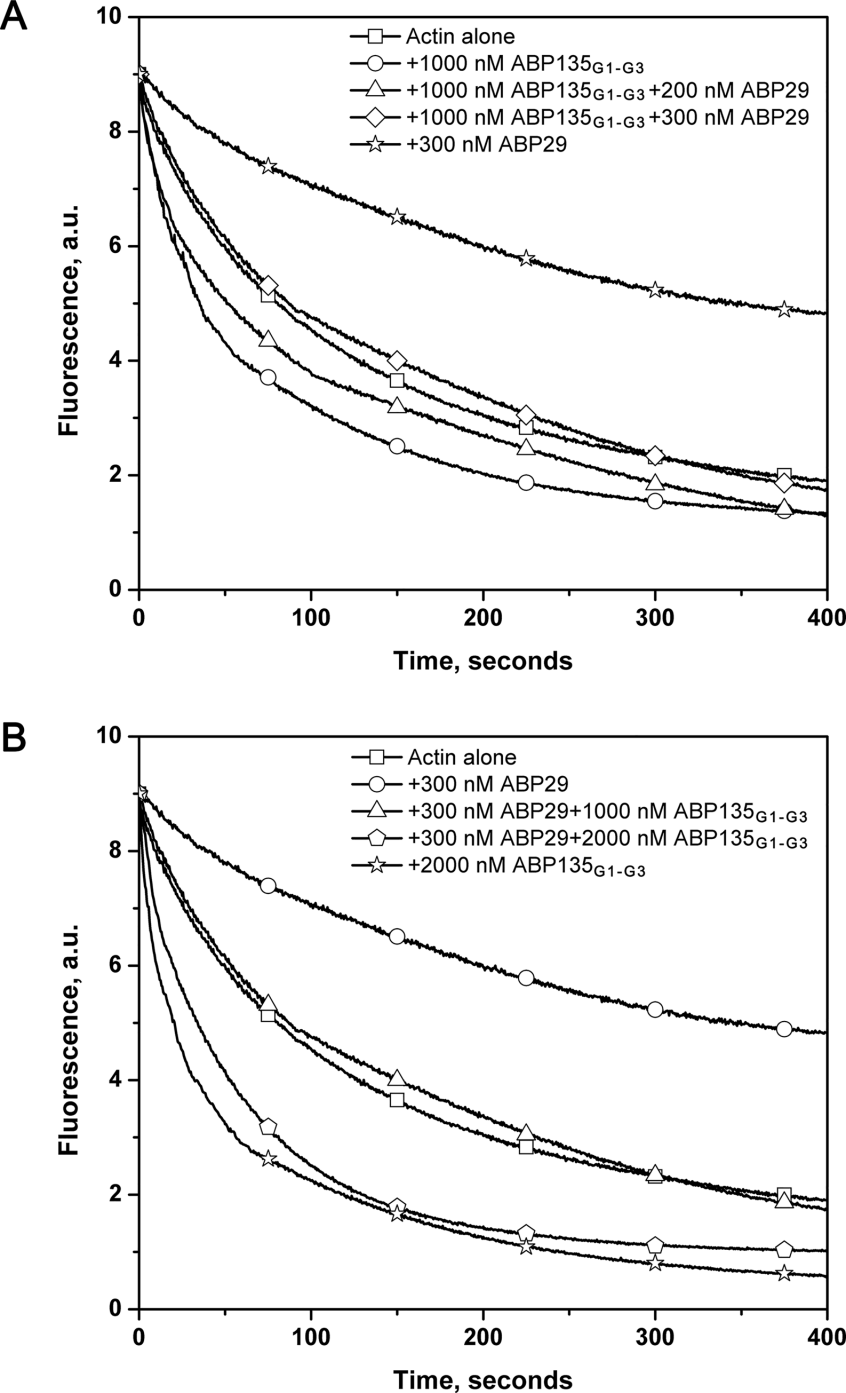
ABP29 and ABP135_G1-G3_ compete with each other in actin depolymerization. (A) Competition experiments between ABP29 and ABP135_G1-G3_ were performed during the F-actin depolymerization process. A fixed amount of ABP135_G1-G3_ (1000 nM) and different amounts of ABP29 (0, 200 nM and 300 nM) were simultaneously incubated with 5 μM F-actin (50% pyrene-labeled). The kinetics of actin depolymerization were monitored by measuring the decrease in pyrene fluorescence intensity. Different combinations of reactions are indicated in the upper right. (B) A fixed amount of ABP29 (300 nM) and different amounts of ABP135_G1-G3_ (0, 1000 nM and 2000 nM) were simultaneously incubated with 5 μM F-actin (50% pyrene-labeled). Different combinations of reactions are indicated in the upper right.

Recent studies have also shown that ABP29 and LdABP41, mRNA-spliced variants of lily ABP135, are expressed in pollen tubes [19,20]. Thus, these proteins may compete in the regulation of actin dynamics. To determine whether and how ABP29 affects ABP135_G1-G3_ activity and vice versa, actin depolymerization assays were conducted. As shown in Fig 7A, when the amount of ABP135_G1-G3_ was fixed (at 1000 nM) and the concentration of ABP29 was varied (0 nM, -200 nM or -300 nM), ABP29 reduced the effects of ABP135_G1-G3_ on F-actin depolymerization. Similarly, when the amount of ABP29 was fixed (at 300 nM) and the concentration of ABP135_G1-G3_ was varied (0 nM, -1000 nM or -2000 nM), ABP135_G1-G3_ effectively reduced the effects of ABP29. Taken together, the presence or deletion of the G3 domain may play a special cooperative role in regulating dynamic actin remodeling in plants.

### ABP29 stabilizes actin by inhibiting F-actin depolymerization during the process of dilution-mediated depolymerization in contrast with typical villin/gelsolin/fragmin superfamily members

**Fig 8:**
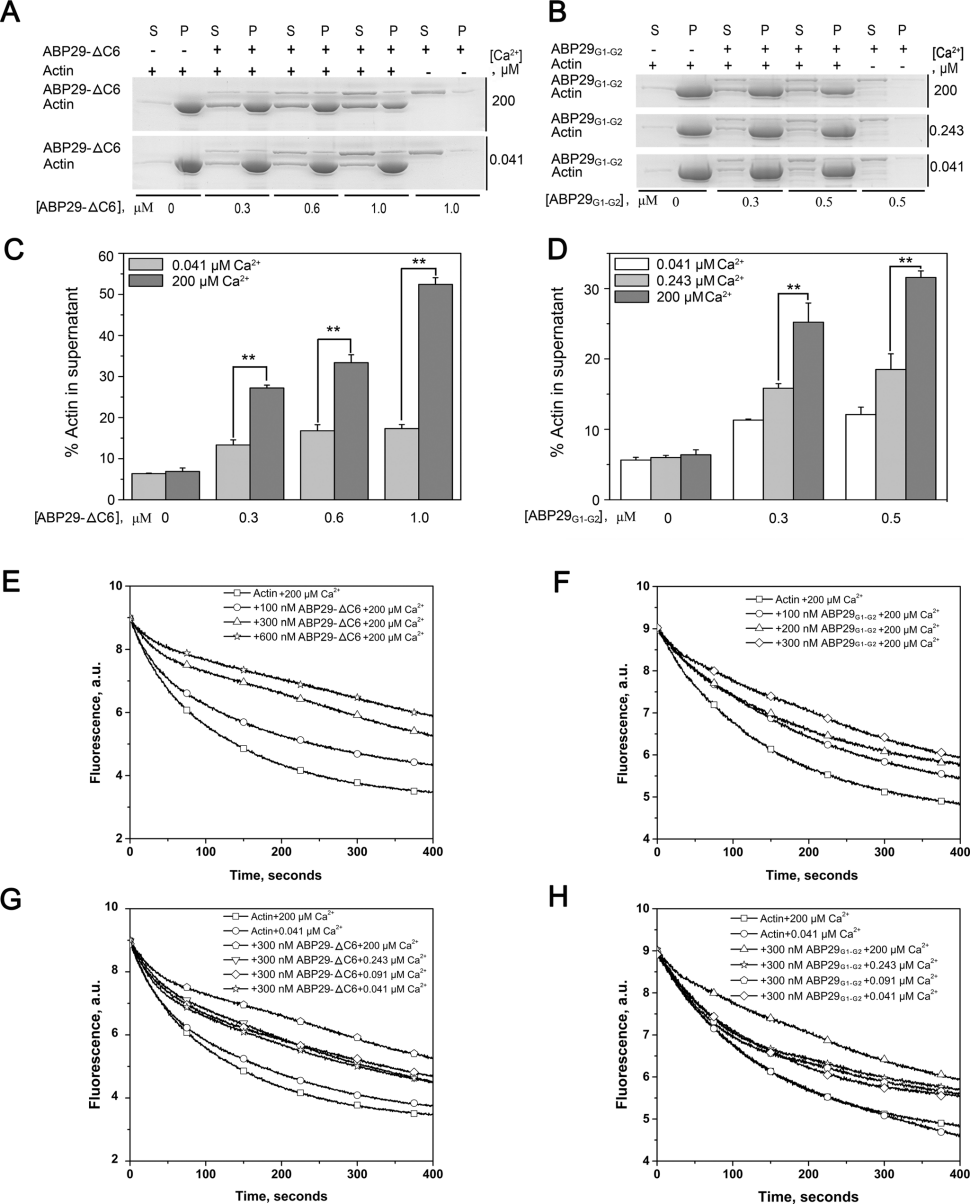
Recombinant ABP29-△C6 and ABP29_G1-G2_ sever and/or depolymerize F-actin and stabilize F-actin *in vitro*. (A) The severing and/or depolymerization activity of ABP29-△C6 exerted on actin filaments was determined using high-speed co-sedimentation assays in the presence of 200 μM and 0.041 μM free Ca^2+^; "(s)" indicates supernatant, and "(p)" indicates pellets. (B) The severing and/or depolymerization activity of ABP29_G1-G2_ exerted on actin filaments was determined using high-speed co-sedimentation assays in the presence of various concentrations of free Ca^2+^ (200, 0.243 and 0.041 μM); "(s)" indicates supernatant, and "(p)" indicates pellets. (C) and (E) Statistical analysis results from (A) and (B), respectively. The error bars represent ± SE (n = 3); **P < 0.01 (Student’s *t* test). (E) and (F) Kinetics of actin depolymerization in the presence of various concentrations of ABP29_G1-G2_ and ABP29-△C6 in the presence of 200 μM free Ca^2+^. The change in pyrene fluorescence accompanying actin depolymerization was monitored. Different combinations of the reactions are indicated in the upper right. (G) and (H) 300 nM ABP29_G1-G2_ or ABP29-△C6, respectively, was incubated with 5 μM F-actin (50% pyrene-labeled) for 5 min in the presence of various concentrations of free Ca^2+^ chelated by EGTA. The kinetics of actin depolymerization were monitored by measuring the change in fluorescence intensity. Different combinations of reactions are indicated in the upper right.

Interestingly, ABP29, which contains the G1 and G2 domains and parts of the G2-G3 linkers of ABP135, inhibits F-actin depolymerization in a Ca^2+^-dependent manner [20]. To elucidate the underlying difference and determine whether the G1-G2 domains of ABP29 alone can enhance actin filament depolymerization, we generated two truncated variants of ABP29. The first was ABP29-△C6, a version of ABP29 in which six C-terminal amino acids of ABP29 were removed (this six amino acid sequence is not included in ABP135). The second was ABP29_G1-G2_, which included only the G1 and G2 domains of ABP29 (Fig 1B). As shown in Fig 8, ABP29-△C6 and ABP29_G1-G2_ severed and/or depolymerized actin filaments, and during the process of dilution-mediated depolymerization, they inhibited F-actin depolymerization in a Ca^2+^-sensitive manner similar to ABP29. We found that the effect exerted by ABP29 on actin depolymerization was the opposite of that of typical villin/gelsolin/fragmin superfamily members, which instead accelerate F-actin depolymerization during the process of dilution-mediated depolymerization.

## Discussion

Pollen tube growth is a vital process in angiosperms and is necessary for double fertilization. Genetic and pharmacological evidence has demonstrated that the actin cytoskeleton plays a vital role in this process; the disruption of actin filaments leads to the retardation of pollen germination and pollen tube growth [20,43–48]. In addition, Ca^2+^ has been shown to exist in the pollen tube in a concentration gradient and to be indispensable for this process [49,50]. For example, within the pollen tube, the Ca^2+^ concentration is 5–10 μM in the tip area but 200 nM in the shank [39–41,49,50]. Actin cytoskeleton dynamics are fundamental to the role of the cytoskeleton in pollen tube growth, which is directly regulated by numerous ABPs [18,51]. Among them, the villin/gelsolin/fragmin superfamily is a major group of Ca^2+^-dependent ABPs and may act as the "sensor" and "executor" of the Ca^2+^ [13,19,30]. Importantly, many members of this family have been identified in pollen and have been shown to be necessary for pollen germination and pollen tube growth, regulating diverse actin structures in various stages and spaces, such as AtVLN5 [38] and AtVLN2 [52] in *Arabidopsis thaliana*, ABP135 [14,15] and ABP115 [16,17] in *Lilium longiflorum*, PrABP80 in *Papaver rhoeas* [13], LdABP41 in *Lilium davidii* [19] and ABP29 in *Lilium longiflorum* [20]. The functions of the proteins in this superfamily are determined by different gelsolin-like domains, which play distinct roles in actin dynamics [1,3,5]. To date, the function of the G3 domain remains unclear. Therefore, elucidating the function of this domain would contribute to our understanding of the mechanism by which members of the villin/gelsolin/fragmin superfamily regulate actin dynamics in pollen. In this paper, we compared differences between ABP29 and ABP135_G1-G3_ in terms of actin dynamics in the presence of different concentrations of Ca^2+^. The results demonstrated that ABP135_G1-G3_ had greater severing and/or depolymerization and nucleating abilities than ABP29 in the presence of high levels of Ca^2+^ (200 and 10 μM) (Figs 1C and 1D, 2 and 3), whereas ABP29 exhibited greater capping activity relative to ABP135_G1-G3_ in the presence of low concentrations of Ca^2+^. These findings may also indicate that ABP29 experiences slower off rates and therefore remains bound to the filament barbed ends to protect them from both depolymerization and elongation. Moreover, ABP29 inhibits F-actin depolymerization in a Ca^2+^-dependent manner during the process of dilution-mediated depolymerization (Fig 6). All of these results suggest that the G3 domain plays a specific role in regulating the activities of villin/gelsolin/fragmin superfamily members in the presence of different Ca^2+^ concentrations.

What is puzzling is that the severing and/or depolymerization activity of ABP135_G1-G3_, which contains the G3 domain, is stronger than that of ABP29 in the presence of high concentrations of Ca^2+^ (such as 200 μM and 10 μM). Although the crystal structures of ABP135_G1-G3_ and ABP29 have not been determined, the structures of full-length gelsolin [5], the G1-G3 domains of gelsolin in complex with G-actin [53,29] and the G4-G6/actin complex [54] and 3D structure of villin [55] have been solved. These excellent prior studies allow us to propose a model to explain the differences in actin severing and/or depolymerization abilities between ABP29 and ABP135_G1-G3_ which reveals that both proteins possess highly conserved gelsolin domains [19,20,56]. The work of Peddada N. and colleagues showed that G1-G2 of gelsolin appears to prefer a collapsed state under Ca^2+^-free conditions and changes to an open shape in 1 mM Ca^2+^, while G1-G3 of gelsolin adopts an open three-lobed and a closed shape under Ca^2+^-free and 1 mM Ca^2+^ conditions, and G1-G3 with mutations in the g2-g3 linker assumes open shape under 1 mM Ca^2+^ conditions [42]. It may be that ABP135_G1-G3_ is a same shape as mutations of G1-G3 of gelsolin. As shown in S2B Fig, in the absence of Ca^2+^,ABP135_G1-G3_ is compact and inactive. According to the work of Burtnick L. D. and coworkers [53,57] as well as the molecular model of ABP135_G1-G3_, the severing steps consist of the following activities: when there are high concentrations of Ca^2+^ (10 μM Ca^2+^), Ca^2+^ binding to the G3 domain of ABP135_G1-G3_ weakens and opens the G1-G3 latch and straightens its long helix. Then, Ca^2+^ binding to the G2 domain further drives the G2–G3 domains towards an active conformation, exposing the buried F-actin binding site in the G2 domain. Finally, the G2 domain binds to the side of the F-actin, and the G1 domain wedges two adjacent actins in the longitudinal axis, causing a cleavage and then leading the G1 domain to bind to the barbed ends simultaneously, resulting in severing. Similarly, although lacking the G3 domain, Ca^2+^ binding to the G2 domain can drive the G1-G2 domains toward a fully active conformation. Then, like ABP135_G1-G3_, the G2 domain binds to the side of F-actin, and the G1 domain wedges two adjacent actins into the longitudinal axis, resulting in severing (S2A Fig). As shown in S2B Fig, in the presence of Ca^2+^, the G2 domain binds to the actin subdomain 2, and the G3 domain may also bind to the actin subdomain 1, which may stabilize the binding activity between the G2 domain and the actin. Because it lacks the G3 domain, the actin severing and/or depolymerization activity of ABP29 is weaker than that of ABP135_G1-G3_. We proposed that the G3 domain may partially contribute to actin severing activity by stabilizing the binding activity between the G2 domain and actin. Furthermore, Selden et al. [58] have proposed that the G1-G3 domains cooperatively sever F-actin. Therefore, ABP135_G1-G3_ may lead to a cooperative severing of F-actin by using two molecules that bind F-actin in close proximity, thereby increasing the severing efficiency.

In general, the villin/gelsolin/fragmin superfamily proteins sever F-actin and then generate more actin ends, thereby accelerating actin depolymerization, as demonstrated by dilution-mediated actin depolymerization assay [1]; however, a capping protein binds to the F-actin ends and thus inhibits actin depolymerization [59]. *In vitro*, ABP135_G1-G3_ and ABP29 exhibit actin severing and/or depolymerization and capping activities; however, ABP29 inhibits F-actin depolymerization, whereas ABP135_G1-G3_ accelerates its depolymerization during the process of dilution-mediated depolymerization. We propose that ABP135_G1-G3_ might preferentially bind to F-actin because the G3 domain stabilizes the interaction between the G2 domain and the F-actin. Otherwise, the capping activity of ABP29 plays a main effect on F-actin for the special structure of ABP29 with G1-G2 domains. Without the latch of the G3 domain, the G1 domain of ABP29 could directly bind to the ends of F-actin, leading to cap the barbed ends after the severing. So ABP29 resulted in more actin in the supernatants and shorter filaments compared to ABP135_G1-G3_ (Figs 1 and 2) and stabilized F-actin during dilution-mediated depolymerization. To the best of our knowledge, thus far, ABP29 is a natural product and the only member of the villin/gelsolin/fragmin superfamily with severing and capping activities that stabilizes F-actin during dilution-mediated depolymerization in a Ca^2+^-dependent manner. We thus obtained two truncated versions of ABP29: ABP29-△C6 and ABP29_G1-2_. As shown in Fig 8, like ABP29 (Fig 1), ABP29-△C6 and ABP29_G1-G2_ sever and/or depolymerize actin filaments and protect F-actin from dilution-mediated depolymerization (Fig 8A and 8B). Because native ABP29 existing in pollen only contains the G1-G2 domains, we speculate that plants may have evolved a special mechanism by which they fulfill specific roles in regulating actin dynamics during this process. Therefore, it would be interesting and helpful to conduct further structural and biochemical analyses of the G1-G2 domains to explore this potential mechanism in plants.

The biochemical activities and expression patterns of the villin/gelsolin/fragmin superfamily members are diverse and distinct during pollen germination and pollen tube growth [30]. For example, in ungerminated lily pollen, only PrABP80, LdABP41 and ABP29 can be found, indicating that these proteins play vital roles in maintaining the actin cytoskeleton in a dot-like form as a storage stage via their actin severing, nucleating and capping activities [30]. After pollen germination, the expression levels of LdABP41 decrease dramatically, and ABP135 and ABP115 specifically emerge [30]. ABP135 and ABP115 may participate in the formation of higher-order actin bundles via their actin severing, bundling, nucleating and capping activities. The expression levels of ABP29 remain stable throughout the process, indicating that it consistently regulates actin dynamics [30]. In this study, we used rabbit muscle actin and lily proteins to perform the experiments because actin is one of the most highly conserved proteins, as it has changed little over the course of evolution [60]. We showed that ABP135_G1-G3_ and ABP29 possess similar actions in terms of actin severing and/or depolymerization, nucleating and capping, but their activities exhibit some differences under different concentrations of Ca^2+^. Moreover, these proteins neutralize activity in the dilution-mediated depolymerization assay, and this difference is specifically attributable to the G3 domain. In conclusion, the presence or absence of the G3 domain may be an important evolutionary mechanism to regulate the activities of gelsolin/fragmin/fragmin proteins, which is necessary for pollen germination and pollen tube growth.

## Supporting Information

S1 Fig ABP29 and ABP135G1-G3 shifted the critical concentration of actin assembly. Pyrene-labeled G-actin (20% labeled) with varying concentrations (0–4 μM) was polymerized alone or with a 1/200 molar ratio of human CapG:actin for 18 h at room temperature in the presence of 0.5 μM ABP29 or ABP135_G1-G3_ in F-buffer, and the final fluorescence intensity of pyrene (excitation at 365nm and emission at 407 nm) was measured.(A) Actin polymerized alone in the presence of 0.5 μM ABP29 or ABP135_G1-G3_ in F-buffer; (B) actin polymerized with a 1/200 molar ratio of human CapG:actin in the presence of 0.5 μM ABP29 or ABP135_G1-G3_ in F-buffer. Linear best fit of the data, and plotted as arbitrary fluorescence units versus actin concentration, was used to determine the intercept with x axis.
(TIF)

S2 Fig The models of ABP29:actin and ABP135_G1-G3_:actin. (A) Model of Ca^2+^-free ABP29 interacting with actin: ABP29 contains the G1 and G2 domains. Actin is colored in gray, with subdomains 1, 2, 3 and 4 indicated. Ca^2+^ ions are depicted as green spheres. (B) Model of Ca^2+^-free ABP135_G1-G3_ interacting with actin. (C) and (D) Models of the active states of ABP29:actin and ABP135_G1-G3_:actin, respectively. Molecular graphics were created with VMD software package. Crystal structure of Ca^2+^-free plasma gelsolin (PDB:1D0N); crystal structures of gelsolin domains G1-G3:actin (PDB:1RGI) and the gelsolin G4-G6/ACTIN complex (PDB:1H1V).
(TIF)
