# Supplementary figures and images for: Gelsolin-Like Domain 3 Plays Vital Roles in Regulating the Activities of the Lily Villin/Gelsolin/Fragmin Superfamily
Source: PLoS One. 2015 Nov 20;10(11):e0143174. doi: 10.1371/journal.pone.0143174 (PMC4654503; doi:10.1371/journal.pone.0143174)

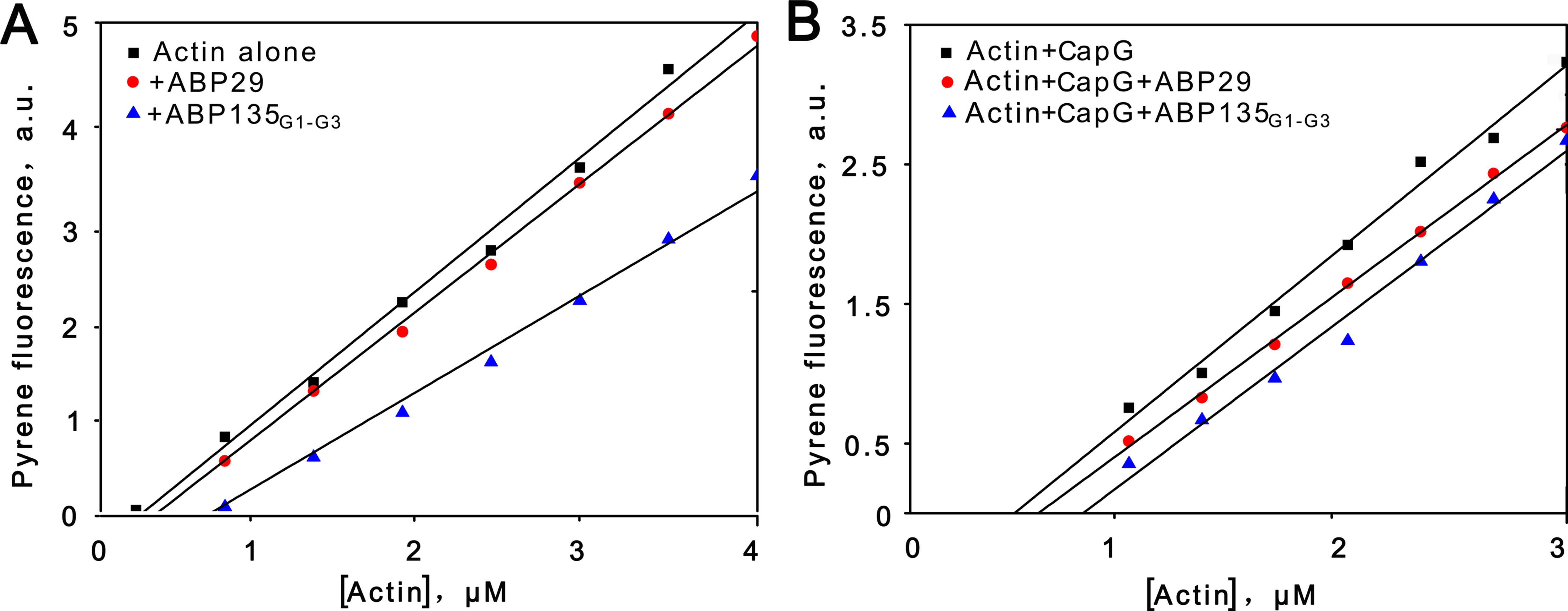

Supplement: S1 Fig — Pyrene-labeled G-actin (20% labeled) with varying concentrations (0–4 μM) was polymerized alone or with a 1/200 molar ratio of human CapG:actin for 18 h at room temperature in the presence of 0.5 μM ABP29 or ABP135G1-G3 in F-buffer, and the final fluorescence intensity of pyrene (excitation at 365nm and emission at 407 nm) was measured.(A) Actin polymerized alone in the presence of 0.5 μM ABP29 or ABP135G1-G3 in F-buffer; (B) actin polymerized with a 1/200 molar ratio of human CapG:actin in the presence of 0.5 μM ABP29 or ABP135G1-G3 in F-buffer. Linear best fit of the data, and plotted as arbitrary fluorescence units versus actin concentration, was used to determine the intercept with x axis. (TIF) [file pone.0143174.s001.tif]

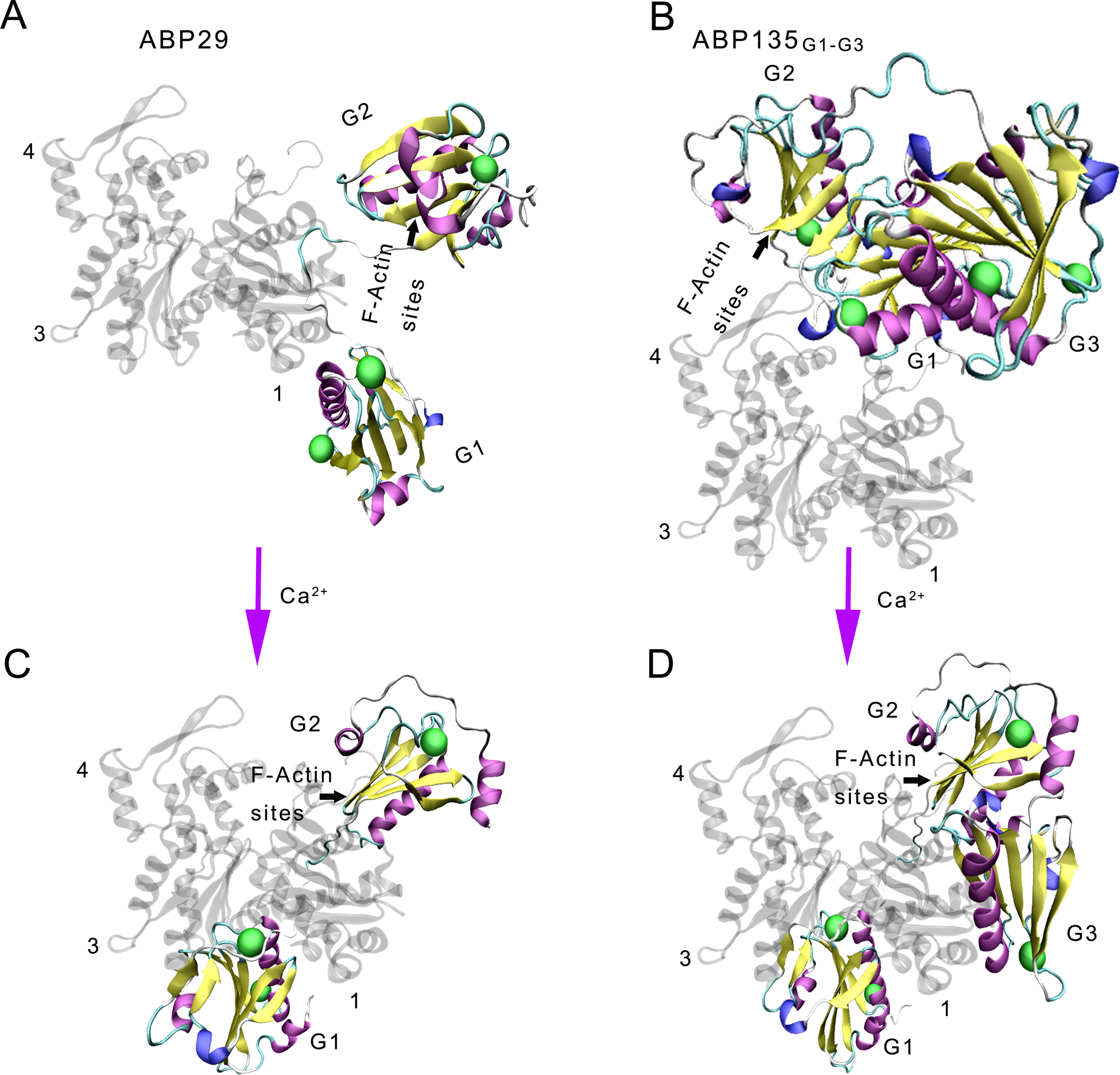

Supplement: S2 Fig — (A) Model of Ca2+-free ABP29 interacting with actin: ABP29 contains the G1 and G2 domains. Actin is colored in gray, with subdomains 1, 2, 3 and 4 indicated. Ca2+ ions are depicted as green spheres. (B) Model of Ca2+-free ABP135G1-G3 interacting with actin. (C) and (D) Models of the active states of ABP29:actin and ABP135G1-G3:actin, respectively. Molecular graphics were created with VMD software package. Crystal structure of Ca2+-free plasma gelsolin (PDB:1D0N); crystal structures of gelsolin domains G1-G3:actin (PDB:1RGI) and the gelsolin G4-G6/ACTIN complex (PDB:1H1V). (TIF) [file pone.0143174.s002.tif]
